# Positive bias in brain and behaviour as a mechanism of transcranial magnetic stimulation depression treatment

**DOI:** 10.1038/s41380-026-03485-8

**Published:** 2026-02-12

**Authors:** Verena Sarrazin, Paulo Suen, Beatriz Cavendish, Marieke Martens, Pedro Henrique Rodrigues da Silva, Anne Britto, Matheus Rassi, Mariana Baptista, Andre R. Brunoni, Jacinta O’Shea

**Affiliations:** 1 Wellcome Centre for Integrative Neuroimaging, University of Oxford, Oxford, OX3 9DU United Kingdom; 2 Department of Psychiatry, Warneford Hospital, University of Oxford, Oxford, OX3 7JX United Kingdom; 3 Oxford Centre for Human Brain Activity (OHBA), University of Oxford, Oxford, OX3 7JX United Kingdom; 4 Departamento e Instituto de Psiquiatria, Hospital das Clínicas da Faculdade de Medicina da USP, São Paulo, Brazil; 5 Oxford Health National Health Service (NHS) Foundation Trust, Warneford Hospital, Oxford, OX3 7JX United Kingdom

**Keywords:** Depression, Predictive markers, Neuroscience, Psychology

## Abstract

Transcranial magnetic stimulation (TMS) is a novel approved therapy for treatment-resistant depression. Little is known about its neurocognitive mechanisms of action. The existing literature has focused on resting-state neuroimaging. It therefore remains unknown what information processing changes TMS induces during treatment that drive mood change. Here we tested the hypothesis that transcranial magnetic stimulation (TMS) treatment changes emotional bias, increasing the focus on positive (versus negative) information processing. 49 patients with major depression received 20 daily sessions of open-label intermittent theta-burst TMS to left dorsolateral prefrontal cortex. Emotional bias was measured using behavioural and functional magnetic resonance imaging tasks of emotional face processing, both at baseline and after eight days of treatment. We tested whether early changes in these measures after the first week predicted clinical outcome at the end of treatment (4 weeks). As predicted, an increase in behavioural and neural measures of positive bias after one week predicted clinical response after four weeks of treatment. Behaviourally, response to TMS treatment was associated with a bias towards interpreting ambiguous facial expressions as positive. Neurally, clinical improvement was related to increased neuroimaging response for the contrast of positive versus negative emotional faces in rostral anterior cingulate cortex (rACC), and a positive bias in task-related functional connectivity between rACC and posterior default mode network. These early neurocognitive changes predicted clinical outcomes after four weeks of treatment, beyond early symptom reduction. Thus, clinical response to TMS treatment was linked to increases in positive bias in emotional processing early during treatment which might represent a neurocognitive mechanism of TMS depression treatment, potentially neurally distinct from antidepressant drugs.

## Introduction

Depression is the leading cause of adult disability worldwide [1]. For the ~30% of patients for whom first-line treatments such as medications are ineffective [2, 3], transcranial magnetic stimulation (TMS) applied to left dorsolateral prefrontal cortex (DLPFC) is an alternative, with around 50%/30% response/remission rates in treatment-resistant patients [4–6]. However, little is known about how TMS treatment works.

Depression is associated with increased focus on negative versus positive information (i.e. cognitive-affective bias) which is theorised to contribute to the development and maintenance of symptoms of depression [7]. Antidepressant treatments (medications, CBT) are hypothesised to act in part by increasing the relative focus on positive versus negative information processing early on during treatment [8–10]. While the neuropsychological theory of antidepressant action was developed based on evidence from medication studies, increased positive bias has been proposed as a common mechanism of action shared across different treatment modalities [7]. To date, this theory has not been tested directly for TMS treatment.

Negative biases in depression are hypothesised to arise from hyper-activation of subcortical regions (amygdala, subgenual cingulate) in response to negative stimuli, accompanied by decreased top-down emotional regulatory control from DLPFC [7]. Resting-state neuroimaging suggests that TMS depression treatment might normalise cortico-limbic dysfunction, however, such studies are conducted without cognitive tasks [11–13], and therefore provide no insight into how information processing changes during treatment. Single-session experiments suggest TMS may alter emotional bias [14–17], however, it is unknown if or how TMS treatment changes emotional processing in a way relevant for clinical outcome.

Key to the neuropsychological theory is that early changes in information processing precede and cause clinical improvement. However, most TMS treatment studies to date have only compared measures at baseline (‘depressed’ state) versus post-treatment (‘non-depressed’ state) (e.g. [12]). Critically, this design is uninformative about the neurocognitive changes induced during treatment that drive mood transition from ‘depressed’ to ‘non-depressed’.

To address this, we tested the hypothesis that TMS-induced increases in behavioural and neural measures of positive bias early during treatment would predict clinical outcome post-treatment. 49 patients with major depressive disorder received open-label TMS treatment of 20 daily sessions of intermittent theta-burst stimulation (iTBS) [5]. Positive bias was measured in emotional face processing using an explicit behavioural task (Facial Expression Recognition Task (FERT) [18]) and an implicit functional magnetic resonance imaging (fMRI) task [19]. Both paradigms have been shown to capture negative biases in depression, and to be sensitive to antidepressant medications [9, 19–23]. Positive bias in the FERT was defined as increased accuracy, speed or response bias in recognising positive versus negative emotional faces. Positive bias in fMRI was defined as increased blood-oxygen-level-dependent (BOLD) response to happy versus fearful faces in the task-positive networks (i.e. positive BOLD response to face stimuli) [24], or task-negative networks (i.e. negative BOLD response) such as the default mode network (DMN)) [25, 26].

## Material and methods

This study has been pre-registered (NCT04969549, https://clinicaltrials.gov/study/NCT04969549 and https://osf.io/rhxm2). A more detailed description of the methods can be found in the Supplementary Material.

### Sample

The **Bra**zil-**En**gland Study on **M**echanisms **a**nd Response **P**redictors of iTBS Treatment (BRAEN-MAP study) was conducted in the Instituto de Psiquiatria do Hospital das Clínicas da Faculdade de Medicina da Universidade de São Paulo between August 2021 and November 2022. Participants were recruited through social media advertisement, Google Ads and flyers posted on bulletin boards at the Hospital das Clínicas. 52 individuals took part in the study (see Supplements for sample size justification). 3 participants discontinued the study after two weeks of iTBS treatment, resulting in a final sample size of 49 participants (Gender: 41 female, 8 male; ethnicity: 7 black, 12 pardo (mixed ethnic ancestry), 27 white, and 2 yellow/Asian (one not declared)). Participants were eligible for the study if they were between 18 and 65 years of age, had a Hamilton Depression Rating Scale (HAM-D) [27] score of 14 or above, and met the criteria for a current diagnosis of major depressive disorder as confirmed by the Mini-International Neuropsychiatric Interview (MINI) [28] (not necessarily treatment-resistant depression). Exclusion criteria were current diagnosis of substance dependence, severe clinical or neurological disorders, suicidal ideation, psychotic symptoms, or manic symptoms (Young Mania Rating Scale > 8). Patients with contraindications to TMS or MRI, such as metallic implants or a diagnosis of epilepsy, were excluded from the study. Treatment-resistant depression (usually defined as at least two unsuccessful prior treatment attempts) was not an inclusion or exclusion criterion in this study. 30 of the participants were taking psychoactive medication (see Supplementary Table 1). Demographic and clinical baseline characteristics are included in Table 1. Participants had experienced an average of 6 depressive episodes (SD = 11.0), with the current episode lasting around 36.5 months (SD = 58.3)(see Figure S12). The study was approved by the National Ethics Committee in Brazil (CAEE: 52384921.6.0000.0068), and all participants gave written informed consent according to the Declaration of Helsinki.

**Table 1:** Baseline characteristics.

	Responders (n = 33)	Non-Responders (n = 16)	Test statistic	*p*-value
Gender				
Female (%)	28 (85%)	13 (81%)	Fisher’s exact test	>0.99
Male (%)	5 (15%)	3 (19%)		
Age in years, mean (SD)	39.9 (10.9)	37.6 (9.2)	*t*(35.2) = −0.80	0.43
HAM-D, mean (SD)	18.3 (2.8)	17.6 (2.3)	*t*(35.6) = −0.96	0.34
MADRS, mean (SD)	24.1 (4.3)	26.0 (5.3)	*t*(25.1) = 1.27	0.21
YMRS, mean (SD)	0.91 (1.1)	0.88 (1.3)	*t*(25.9) = −0.09	0.92
PANAS				
Positive, mean (SD)	15.7 (3.9)	14.9 (3.1)	*t*(34.5) = −0.76	0.45
Negative, mean (SD)	29.2 (6.7)	31.2 (7.5)	*t*(24.5) = 0.89	0.38
STAI				
Trait, mean (SD)	64.5 (7.4)	61.0 (7.4)	*t*(31.9) = 1.1	0.27
State, mean (SD)	66.9 (6.9)	63.0 (8.0)	*t*(27.6) = 0.82	0.41
Use of psychoactive medication				
No. taking medication (%)	19 (58%)	11 (69%)	Fisher’s exact test	0.54
No. not taking medication (%)	14 (42%)	5 (31%)		

Welch two-sample t-tests and Fisher’s exact tests were conducted to test for baseline differences between Responders and Non-Responders. All were non-significant.

*HAM-D* Hamilton Depression Rating Scale, *MADRS* Montgomery-Asberg Depression Rating Scale [63], *YMRS* Young Mania Rating Scale [64], *PANAS* Positive and Negative Affect Schedule [65], *STAI* and State-Trait Anxiety Inventory [66].

### Procedure

**Fig. 1:**
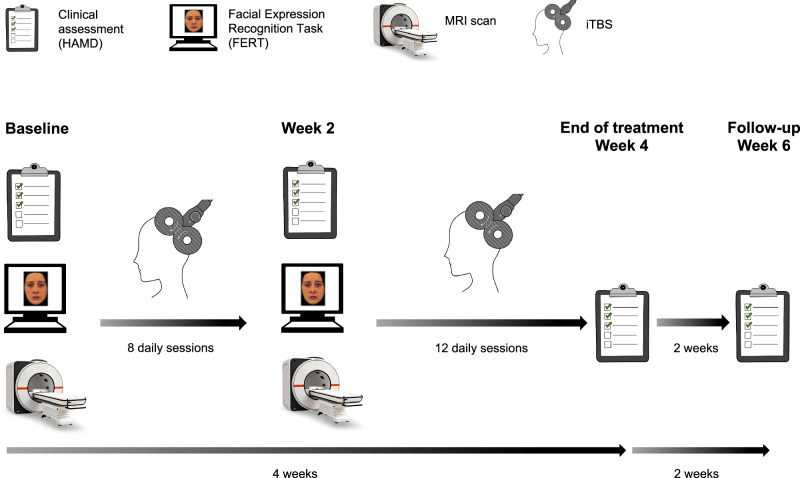
Experimental procedure. Participants completed a Baseline experimental session involving clinical assessments, the Facial Expression Recognition Task, and an MRI scan (including task fMRI). After ~8 daily iTBS sessions, the second experimental session (Week 2) was conducted which was identical to the Baseline session. Participants received a total of 20 iTBS sessions over 4 weeks. Clinical assessments were obtained weekly throughout the treatment period, including at the end of treatment (Week 4), and at follow-up (Week 6). The primary outcome measure timepoint was end of treatment (Week 4). Figure adapted from [67].

Participants were invited to a ‘Baseline’ experimental session which included clinical assessments, a behavioural task (Facial Expression Recognition Task (FERT)), and an MRI scan, including a structural T1 scan and task fMRI (Fig. 1). After completion of the Baseline session, participants started iTBS treatment. TMS treatment consisted of 20 sessions of iTBS delivered over the course of four weeks (Monday to Friday). The protocol included 1,800 pulses per session applied at 100% resting motor threshold. The resting motor threshold was defined as the lowest intensity required to elicit a contraction in the first dorsal interosseous muscle in 3 out of 5 TMS pulses over the motor hotspot. The resting motor threshold was assessed on the first two days of each treatment week. If there was a significant change of more than 5 percentage points between the two days, the motor threshold was reassessed on the third day (or even further), until there was no significant variation from one day to the next.

After around 8 iTBS sessions, participants completed a second experimental session (‘Week 2’) identical to the Baseline session. Due to practical considerations, the exact number of iTBS sessions varied between 7 and 10 which was controlled for in the analysis (4/37/4/4 participants received 7/8/9/10 iTBS sessions before the second experimental session). Participants completed the clinical assessments again at the end of treatment (Week 4), and at a two-week follow-up (Week 6).

### Clinical outcome measures

The primary outcome measure was the change in Hamilton Depression Rating Scale (17-item version)(HAM-D) [27] score from Baseline to the end of treatment (Week 4). *Clinical Response* was defined as a categorical variable reflecting a reduction in HAM-D score from Baseline to Week 4 by at least 50% (‘Responders’), or less than 50% (‘Non-Responders’). To increase statistical power [29], HAM-D change was also coded as a continuous variable, *HAM-D Improvement* (i.e. Baseline HAM-D – Week 4 HAM-D, a positive value indicates symptom reduction). The difference in HAM-D scores (rather than percentage change in HAM-D scores) was used due to statistical advantages [30] (Baseline HAM-D scores were controlled for, see analysis section).

The HAM-D score at Week 4 was missing for one participant (Week 6 score was available). Reduction in HAM-D score at Week 4 was estimated by fitting an exponential decay function to the remaining HAM-D reduction scores for this participant (Week 0–3).

### Facial expression recognition task (FERT)

The FERT was adapted from Young et al. [18]. The task protocol included 250 trials in total, separated into two blocks of 62 and two blocks of 63 trials. On each trial, a face with an emotional expression was presented for 500 ms. Participants were instructed to indicate the emotion of the face as quickly and accurately as possible by clicking onto the corresponding key on a labelled keyboard. Emotions included happy, surprise, anger, fear, sadness, disgust and neutral. Each emotion (apart from neutral) was presented at 10 different intensities, by four different actors (i.e. 4 ×10 images per emotion). Ten neutral faces were included as well.

As outcome measures, four Positive Bias measures were defined based on different performance metrics. These performance metrics were calculated separately for positive (happiness, surprise) and negative emotions (fear, anger, sadness, disgust). Positive Bias measures were defined as the ratio of each performance metric for positive vs. negative stimuli, and were log-transformed to ensure symmetry and interpretability. All Positive Bias measures were defined in a way so that a higher value indicates a more positive bias.

#### Positive bias unbiased hit rate

The Unbiased Hit Rate (based on signal-detection theory [31]) is a measure for how accurately participants identified each emotion. It takes into account how often each emotion was correctly identified, but also how often each emotion was chosen incorrectly:

$$	{Unbiased}\,{Hit}\,{Rate}=\\ 	{percentage}\,{emotion}\,{correctly}\,{identified}* \,\frac{{number}\,{of}\,{trials}\,{emotion}\,{correctly}\,{chosen}}{{number}\,{of}\,{trials}\,{emotion}\,{chosen}}$$

The measure of interest was *Positive Bias Unbiased Hit Rate*, defined as the log-transformed ratio of Unbiased Hit Rate averaged across positive emotions (happy, surprise) divided by averaged Unbiased Hit Rate for negative emotions (fear, sadness, anger, disgust).

#### Positive bias reaction time

For each emotion, the median reaction time was calculated by taking the median across all trials in which the emotion was correctly identified. *Positive Bias Reaction Time* was calculated as the log-transformed ratio of the median reaction time for negative emotions divided by the median reaction time for positive emotions.

#### Positive bias efficiency

*Efficiency* combines accuracy and speed into one measure, by dividing *Unbiased Hit Rate* by the median reaction time. Positive Bias Efficiency was defined as the log-transformed ratio of *Efficiency* for positive emotions divided by *Efficiency* for negative emotions.

#### Positive bias misclassification

The number of Misclassifications for each emotion was the number of times the emotion has been chosen incorrectly. *Positive Bias Misclassification* was defined as the log-transformed ratio of Misclassifications as positive emotions, divided by the number of Misclassifications as negative emotions (this is independent of whether the correct emotion was positive or negative). In contrast to the above measures which capture a bias in the ability to recognise emotions, this measure captures a bias in the response criterion (i.e. a general tendency to indicate a positive or negative emotion), and has been found to be sensitive to the effects of antidepressant medication [21].

FERT data were analysed in R (version 4.1.1) [32]. To test whether changes in positive bias were related to Clinical Response, an ANOVA was conducted for each positive bias measure as dependent variable, Time (Baseline vs. Week 2) as within-subject factor and Clinical Response (Responders vs. Non-Responders) as between-subject factor. Significant findings were cross-validated using a logistic regression analysis predicting Clinical Response. Change in positive bias was included as regressor of interest, and Baseline HAM-D, Age, Gender, use of Psychoactive Medication (yes/no) and Number of iTBS Sessions (before ‘Week 2’ time point) as control regressors. Significant interaction effects of Clinical Response and Time were followed up using an ANOVA to test whether the effect was driven by a specific emotion (change in positive bias as dependent variable, Emotion (Happiness, Surprise, Fear, Anger, Sadness, Disgust) and Time as within-subject factors, and Clinical Response as between-subject factor).

To test whether changes in positive bias might predict HAM-D Improvement as continuous variable, linear regression analyses were performed separately for each positive bias measure, using HAM-D Improvement as dependent variable, and change in positive bias as predictor. Baseline HAM-D, Age, Gender, Psychoactive Medication and Number of iTBS Sessions were included as control regressors.

Reported p-values are either uncorrected (‘*p*’), or corrected for multiple comparisons (four comparisons since there were four positive bias metrics) using Bonferroni correction (‘*p*_*corr*_*’*).

### Emotional faces fMRI task

Participants completed a gender discrimination task which involved passive processing of emotional facial expressions [19]. The task included four blocks of happy faces and four blocks of fearful faces, which were interleaved by nine blocks displaying only a fixation cross. In total, 120 fearful and 120 happy faces were presented for 100 ms each. Participants were instructed to report the gender of the presented face via button press. Although the emotional expression of the faces is irrelevant to the task, the task has been shown to activate emotional processing networks [9, 19].

For each participant, the median reaction time (RT) was calculated separately for happy faces and fearful faces (correct responses only). Accuracy was defined as the percentage of correct responses for happy or fearful faces. RT and accuracy were analysed in repeated-measures ANOVAs, including Session, Emotion and Clinical Response as independent variables (see Supplementary Material). Moreover, regression analyses were run to predict HAM-D Improvement, including RT or accuracy as regressor of interest, as well as Baseline HAM-D, Age, Gender, Psychoactive Medication and Number of iTBS Sessions (before the second experimental session) as control regressors.

### FMRI analysis

FMRI data were analysed using FSL (FMRIB Software Library) [33]. Data and task design were modelled using General Linear Models (GLM) in FEAT (FMRI Expert Analysis Tool) by convolving a gamma hemodynamic response function with the task regressors for the presentation of happy and fearful faces. The temporal derivatives were added as additional regressors. On the first level, the main contrast of interest was Happy > Fearful, but the constituent contrasts Happy > Fixation and Fearful > Fixation were analysed as well. As additional method for motion correction, each individual’s motion parameter estimates from MCFLIRT were included in the GLM (6 additional regressors for rotation or translation). On the second level, a fixed-effects analysis was run for each individual, to assess increase in BOLD response from Baseline to Week 2 (Week 2 > Baseline).

On the third level, data from all participants were combined in a mixed-effects analysis to test for relationships between increase in positive bias from Baseline to Week 2 and clinical outcome at Week 4. Changes in positive bias might be observed in task-positive networks (positive BOLD response) or task-negative networks (negative BOLD response). We refer to increases in de-activations (i.e. more negative BOLD signal) in task-negative networks as ‘increase in negative BOLD response’ (this terminology was adopted from Grimm et al. [34]). Please note that an increase in positive bias can also be interpreted as change in negative bias in the opposite direction (e.g. an increase in Happy > Fear is equivalent to a decrease in Fear > Happy).

Statistical inference was performed using Randomise (FSL’s tool for nonparametric inference) [35] and threshold-free cluster enhancement (TFCE) [36] controlling the family-wise error rate at 0.05. The GLM included HAM-D Improvement as continuous regressor to test for correlations with changes in brain activity.

Additional analyses were run including Clinical Response as categorical regressor (instead of HAM-D Improvement), which yielded similar results (see Supplementary Material and Supplementary Figure S5). All analyses included Baseline HAM-D, Age, Gender, Psychoactive Medication and Number of iTBS Sessions (before the second fMRI scan) as control regressors of no interest. All regressors were de-meaned.

In addition to whole-brain analyses, region-of-interest (ROI) analyses were performed for our a priori ROIs (left and right amygdala, left and right insula, ACC, ventral striatum). Small volume correction analyses were performed using anatomical masks based on the Harvard-Oxford Structural Atlas in FSL (threshold = 50%, i.e. all voxels with a probability of belonging to the ROI of at least 50%) based on the GLMs described above. The mask for the ventral striatum was derived from the Oxford-GSK-Imanova Structural–anatomical Striatal Atlas. Significance within the anatomical masks was assessed using TFCE and Randomise. To correct for multiple comparisons across the six ROIs, the alpha level was set to 0.05/6 = 0.0083 (maps thresholded at 1-*p* = 0.9917).

To visualise the results, individual parameter estimates for significant clusters were extracted from the first-level statistical maps (using Featquery).

To further investigate our main finding, a correlation between Clinical Improvement and increase in negative BOLD response for Happy > Fear in the rostral ACC, we conducted a psycho-physiological interaction (PPI) analysis [37] using the significant cluster in the rostral ACC as seed region. A binary mask was created based on the significant cluster in the rostral ACC. This mask was warped into individual participant space. The *Fslmeants* tool was used to extract the timecourse of the signal within the mask for each participant and each session. The design matrix on the first level included regressors for the presentation of fearful and happy faces, one regressor representing the mean timecourse extracted from the rostral ACC mask, a PPI regressor for happy faces (interaction between Happy regressor and timecourse extracted from the rostral ACC mask), and a PPI regressor for fearful faces (interaction between Fearful regressor and timecourse extracted from the rostral ACC mask). The contrast of interest was Happy > Fearful, i.e. brain regions that show higher connectivity with rostral ACC during the presentation of happy compared to fearful faces (i.e. positive bias in connectivity). In line with the task fMRI analysis described above, the second level combined Baseline and Week 2 sessions for each participant to calculate the change between them (Week 2 > Baseline). On the third-level, group-level statistics were evaluated using Clinical Improvement as regressor of interest. The analysis included Baseline HAM-D, Age, Gender, Psychoactive Medication and Number of iTBS Sessions (before the second fMRI scan) as control regressors of no interest.

To ensure that our results were not confounded by changes in motion between sessions or groups, we analysed whether estimates of absolute or relative motion changed over time and whether there was a relationship with clinical outcome. There was no evidence for such potential confounds (see Supplementary Material, Supplementary Figure 10).

### Regression analysis and elastic net regression

To test whether the change in positive bias measures might predict HAM-D Improvement beyond early HAM-D change and demographic variables, two elastic net regression models were trained in Python (version 3.11.3) using *Scikit-Learn* (version 1.5.2). The first model included early HAM-D change (Week 2), Baseline HAM-D, Age, Gender and Psychoactive Medication as predictor variables. The second model additionally included the change in all positive bias measures which were found to be related to clinical outcome. The models were trained and evaluated in a nested cross-validation procedure using the *R*^*2*^ score as evaluation metric (see Supplements). To compare the fit of the two models while controlling for the number of regressors, we calculated the Akaike Information Criterion (AIC) [38] and Bayesian Information Criterion (BIC) [39], adjusting the degrees of freedom for regularisation [40, 41].

## Results

### Depression scores improved over the course of TMS treatment

**Fig. 2:**
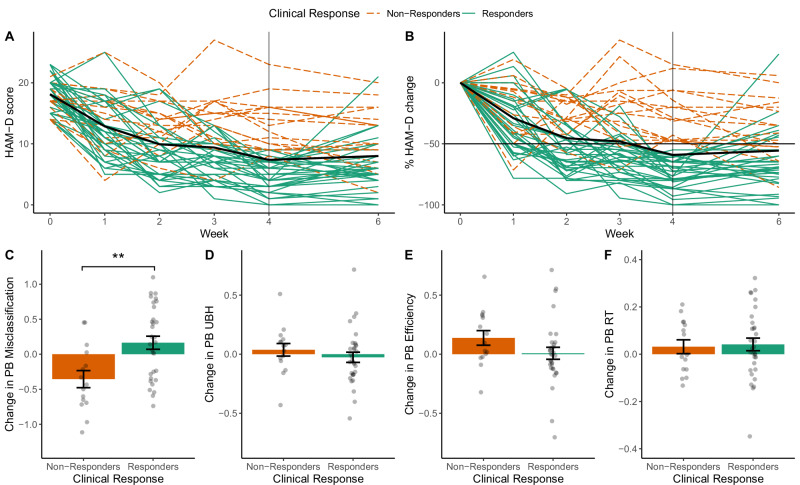
Changes in HAM-D score and in positive bias measures over the treatment period. **A,** **B** Time course of HAM-D scores from Baseline (Week 0) to end of treatment (Week 4) and at follow-up Week 6. **A** Raw HAM-D scores. **B** HAM-D scores as percentage change from Baseline (i.e. 100 * (HAM-D – Baseline HAM-D) / Baseline HAM-D). A negative value indicates a reduction in HAM-D score over time. The main outcome variable was the change in HAM-D from Baseline to Week 4 (marked by a vertical line). Based on percentage reduction in HAM-D score from Baseline to Week 4, 33 participants were classified as Responders (≥50% reduction in HAM-D score), and 16 as Non-Responders. The thick black line represents the group average. **C-F** Change in positive bias measures from the FERT from Baseline to Week 2 as a function of Clinical Response. Responders showed a relatively larger increase in Positive Bias Misclassification than Non-Responders, i.e. they showed an increased tendency to interpret ambiguous facial expressions as positive (**C**). All positive bias measures were defined as log-transformed ratios (see Methods), the y-axis in **C-F** shows the changes in these log-transformed ratios from Baseline to Week 2. A positive value indicates an increase in positive bias over time. ** indicates 0.001 < *p* < 0.01. PB = Positive Bias, RT = Reaction Time, UBH = Unbiased Hit Rate. Error bars represent the standard error of the mean (SEM).

Figure 2 shows mood change over time (Supplementary Table 2). 33 patients were classified as Responders and 16 as Non-Responders.

### Clinical response is associated with positive bias in classification of emotional faces

To assess whether early changes in the FERT were related to clinical outcome, we assessed positive bias in four different performance metrics. The only significant metric was Positive Bias Misclassification which indexes response criterion, i.e. a greater tendency to indicate a positive emotion (rather than a negative one). This measure changed from Baseline to Week 2 as a function of Clinical Response (Clinical Response x Session interaction: *F*(1,44) = 10.5, *p* = 0.002, *p*_*corr*_ = 0.009). Responders showed a relative increase in Positive Bias Misclassification compared to Non-Responders (*Cohen’s d* = 1.31, 95% CI = [0.85, 1.76]; Fig. 2, Supplementary Figure 1, Supplementary Table 3), i.e. they misclassified more faces as positive. A logistic regression analysis confirmed that early change in Positive Bias Misclassification predicted Clinical Response at the end of treatment, after controlling for Baseline HAM-D, Age, Gender, Psychoactive Medication and Number of iTBS Sessions (Clinical Response: *β* = 2.18, *z* = 2.61, *p* = 0.009, *p*_*corr*_ = 0.036, Odds Ratio = 3.39, 95% CI = [1.5, 9.7]). Follow-up tests suggested that this effect was not driven by a change in a particular emotion (see Supplements). The change in Positive Bias Misclassification did not significantly predict HAM-D Improvement as a continuous outcome measure (although there was a positive relationship on a descriptive level which is line with the increase in positive bias in Responders vs. Non-Responders: *β* = 2.3*6*, *t*(39) = 1.67, *p* = 0.103). Change in the remaining positive bias measures was not related to Clinical Response (Clinical Response x Session: *Reaction Time F*[1, 44] = 0.05 *p* = 0.82; *UBH F*(1,44) = 0.77, *p* = 0.39; *Efficiency F*(1,44) = 2.3, *p* = 0.13) or HAM-D Improvement (Supplementary Figure 2).

### Clinical improvement is associated with positive bias in neural response to emotional faces

**Fig. 3:**
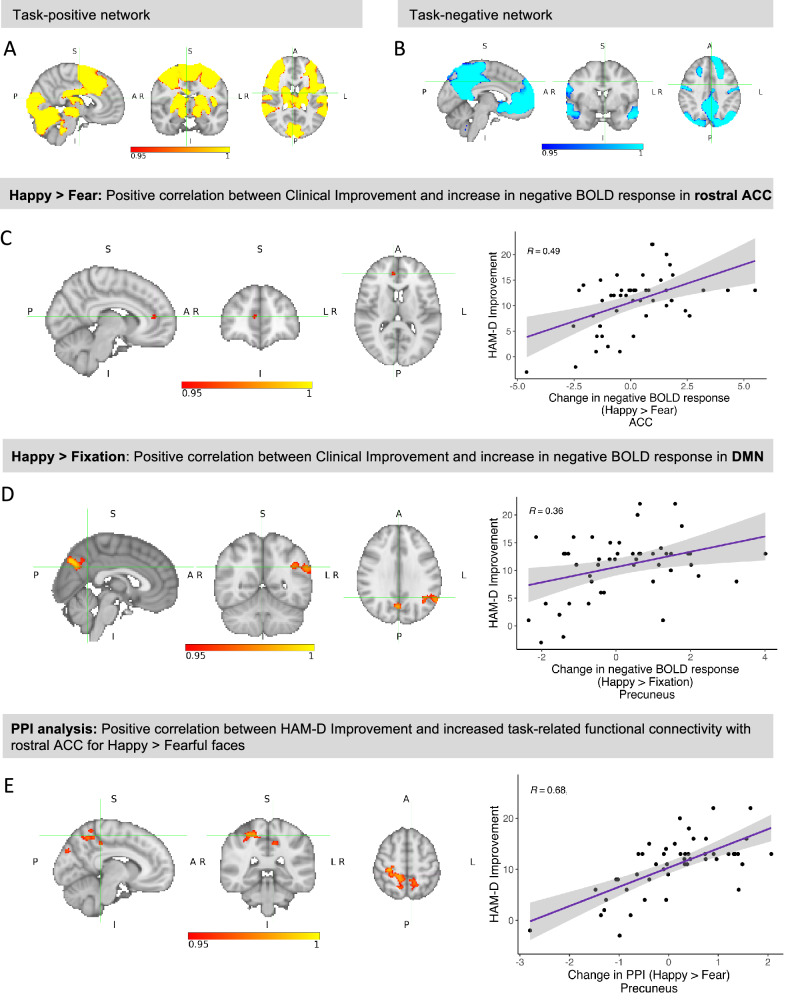
Associations between clinical improvement and early changes in task-related brain activity and connectivity. Group-average task activation: **A** Task-positive network (mean BOLD response to happy and fearful faces). The task activated a single large cluster spanning various areas across the brain (cluster size: 82,062 voxels). The maximum was located in the left temporal occipital fusiform cortex (*tmax* = 16.5, x = −38, y = −50, z = −22, *p* < 0.001). **B** Task-negative network (negative mean response to happy and fearful faces). Seven clusters showed a negative BOLD response during task performance, including medial prefrontal cortex, rostral ACC, precuneus, posterior cingulate and angular gyrus. The maximum was located in the left lateral occipital cortex (*tmax* = 13.9, x = −40, y = −70, z = 34, *p* < 0.001). **C** In the Happy > Fear contrast (whole-brain), HAM-D Improvement correlated positively with an increase in negative BOLD signal in a single cluster in the rostral ACC which overlapped with the task-negative network shown in (B)(17 voxels, *tmax* = 5.35, x = 8, y = 44, z = 12, *p* = 0.045). **D** In the Happy > Fixation contrast, HAM-D Improvement correlated with an increase in negative BOLD response in 3 clusters overlapping the task-negative network, including left precuneus, angular gyrus, and supramarginal gyrus (maximum in the precuneus: *tmax* = 5.70, x = 2, y = −74, z = 38, *p* = 0.008). Please note that a positive value for ‘change in negative BOLD response’ indicates an increase in negative BOLD response (i.e. more negative BOLD signal). **E** A psycho-physiological interaction (PPI) analysis with rostral ACC as seed region revealed a positive correlation between HAM-D Improvement and change in PPI in the Happy > Fear contrast in various areas of the DMN, sensorimotor and visual areas. The peak voxel was located in the right precentral gyrus (*tmax* = 4.78, x = 26, z = −24, z = 60, *p* = 0.014). This suggests that individuals with larger HAM-D Improvement showed a relative increase in connectivity between those areas and rostral ACC when happy faces were presented, and a relative decrease in connectivity when fearful faces were presented, i.e. an increase in positive bias in connectivity. The plot on the right shows the correlation between HAM-D Improvement and increase in PPI for happy vs. fearful faces in the largest clusters spanning the left and right precuneus, precentral gyrus, left posterior cingulate, and right postcentral gyrus.

In the fMRI paradigm, emotional faces (Happy + Fearful) elicited a positive BOLD response across large areas of the brain, including ACC, insula and amygdala (Fig. 3A), and a negative BOLD response in areas of the default mode network (e.g. precuneus and medial prefrontal cortex) (Fig. 3B, Supplementary Table 4).

To test the hypothesis that an early increase (Week 2 – Baseline) in positive bias (Happy > Fear) would be associated with later clinical improvement, we ran a third-level group analysis of Emotion x Time, including HAM-D Improvement as a continuous regressor (controlled for Baseline HAM-D scores). In the whole-brain analysis, HAM-D Improvement correlated positively with an increase in negative BOLD response (i.e. increased *de-activation* [34]) in the Happy > Fear contrast over time (Week 2 – Baseline) specifically in rACC, i.e. with an increase in positive bias (17 voxels, *tmax* = 5.35, x = 8, y = 44, z = 12, *p* = 0.045) (Fig. 3C). ROI analyses did not reveal any further correlations with HAM-D Improvement.

To better understand what was driving this correlation with Happy > Fear, we further analysed the constituent contrasts Happy > Fixation and Fear > Fixation. In the whole-brain analysis, HAM-D Improvement correlated with an increase in negative BOLD response over time (Week 2 – Baseline) for Happy > Fixation in three clusters overlapping the task-negative network, including left precuneus, angular gyrus, and supramarginal gyrus (maximum in the precuneus: *tmax* = 5.70, x = 2, y = −74, z = 38, *p* = 0.008) (Fig. 3D, Supplementary Figure 4, Supplementary Table 5). This is consistent with the findings above and suggests that the increase in positive bias might extend beyond the rACC to other areas of the DMN. No significant clusters emerged in the Fear > Fixation contrast, or in the ROI analyses.

### Clinical improvement is associated with positive bias in task-related connectivity

To further investigate our main finding - the correlation between HAM-D Improvement and increase in negative BOLD response for Happy > Fearful faces in rACC - we conducted a psycho-physiological interaction (PPI) analysis [37]. PPI analysis identifies brain areas which show an increase in functional connectivity (i.e. communication) with a selected seed region in a task-specific psychological context. We ran a whole-brain PPI analysis based on the Happy > Fear contrast using the significant cluster in rACC as seed region. This reveals regions which showed a stronger correlation with rACC during the presentation of happy compared to fearful faces (i.e. positive bias in connectivity).

This revealed a positive correlation between HAM-D Improvement and increase in PPI in the Happy > Fear contrast over time (Week 2 – Baseline) in several regions of the DMN (precuneus, posterior cingulate), sensorimotor areas and visual areas (Fig. 3E, Supplementary Figure 6, Supplementary Table 6). This indicates that individuals with larger HAM-D Improvement showed a relative increase in connectivity between the identified areas and rACC when happy faces were presented, and a relative decrease in connectivity when fearful faces were presented, i.e. an increase in positive bias in connectivity.

### Clinical improvement is associated with positive bias in the fMRI gender discrimination task

Regarding fMRI task performance, an increase in reaction time (RT) for happy faces from Baseline to Week 2 significantly predicted HAM-D Improvement (*β* = 0.015, *t*(35) = 2.03, *p* = 0.0491)(Supplementary Figure 8). An increase in RT for happy faces could indicate attentional capture, i.e. the happy facial expressions might distract participants from the task (discriminating the gender of faces), reflecting increased positive bias. Consistent with this, the RT increase for happy faces correlated positively with the neural increase in response to happy faces in the precuneus (*r* = 0.4, *t*(40) = 2.7, *p* = 0.0083), and with the increase in connectivity (PPI) with rACC for happy vs. fearful faces in the precuneus (*r* = 0.41, *t*(40) = 2.8, *p* = 0.0073) and cuneus (*r* = 0.5, *t*(40) = 3.6, *p* = 0.0007) (Supplementary Figure 9).

### Neurocognitive changes predict HAM-D Improvement beyond early symptom reduction

**Fig. 4:**
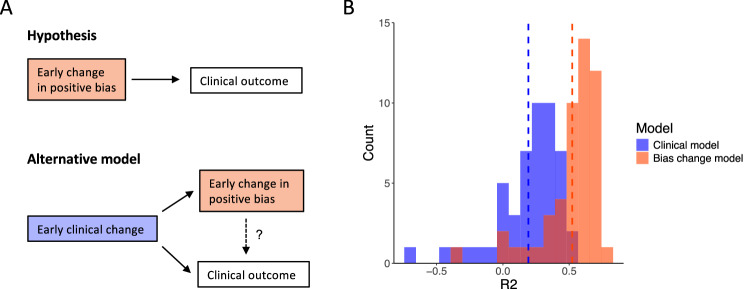
Changes in positive bias measures predicted HAM-D Improvement beyond early clinical change. **A** The neurocognitive theory suggests that early neurocognitive changes drive clinical recovery. An alternative interpretation of our findings might be that early positive bias change might be an effect of early clinical change and unrelated to clinical outcome at the end of treatment. To rule out this possibility, we trained two machine learning models (elastic net regression) to test whether early change in positive bias might predict clinical outcome beyond early clinical change. **B** A ‘Clinical’ model was trained on demographic and clinical data including the early change in HAM-D score (Week 2). Adding the change in behavioural and neural markers of positive bias to the model significantly improved prediction (R^2^ of 0.52 compared to 0.19). This speaks against the alternative model and supports the hypothesis of a causal relationship between early neurocognitive change and clinical outcome.

The analyses reported above assessed which changes in brain and behaviour observed after 10 days of TMS treatment were associated with the later clinical outcome. However, since HAM-D scores had already improved by Week 2 it is unclear whether the cognitive changes preceded and caused clinical improvement. Alternatively, the changes in positive bias might simply reflect early symptom improvement (Fig. 4A). If so, early changes in positive bias measures should *not* predict clinical improvement beyond early HAM-D reduction.

The aim of the last part of the analysis is therefore to test whether early changes in positive bias measures predict Clinical Improvement beyond early HAM-D reduction. In contrast to the analyses reported above, this analysis includes changes in positive bias measures as predictors, and clinical improvement as outcome variable (in the analyses above, Clinical Improvement was included as independent variable, and change in positive bias measures as outcome variable, since this is the standard implementation in fMRI analysis software). Importantly, this last analysis controls for the early clinical change at Week 2 (please note that the HAM-D score at Week 2 is highly correlated with Clinical Improvement; inclusion of the HAM-D score at Week 2 as an additional regressor in the analyses reported above would decrease statistical power to detect a relationship between HAM-D Improvement and changes in brain activity).

We tested this hypothesis by training two machine learning models (elastic net regression, see Methods): one included the early change in HAM-D and baseline demographic data as predictors, the second one additionally included the change in positive bias measures found to be significantly related to clinical improvement in the previous analyses. The aim of this analysis was to test whether the positive bias measures might explain additional variance beyond the early clinical change (not to interpret the actual predictive accuracy). Adding the positive bias measures improved the variance explained (R^2^ score) in left-out data from 19% (SD = 24%) to 52% (SD = 22%) (Fig. 4B, Supplementary Figure 10). AIC and BIC scores both indicated that the more complex model (i.e. the one including the positive bias measures) provided a better fit to the data, even when controlling for the number of regressors, showing that the positive bias measures did indeed explain additional variance (which did not just result from a higher number of regressors) (Clinical model: AIC = 332.9, BIC = 336.7; Bias change model: AIC = 310.7, BIC = 320.2; lower values indicate better fit). This demonstrates that early changes in positive bias explain variance in treatment outcome over and above early HAM-D change. While this analysis does not provide causal evidence, it suggests that early neurocognitive changes are unlikely to simply represent an effect of early clinical change.

## Discussion

The goal of this open-label iTBS treatment study was to test for the first time whether TMS depression treatment increases behavioural and neural markers of positive bias early on during treatment, and whether these changes predict clinical outcome at the end of treatment. Behaviourally, clinical response was related to an increased tendency to interpret facial expressions as positive. Neurally, clinical improvement was associated with increased positive bias in task-related default mode network activity and connectivity. These neural changes predicted clinical outcomes beyond early clinical improvement.

Negatively biased information processing is theorised to contribute to the development and maintenance of depression [7]. Using the FERT to assess biases towards positive or negative facial expressions, we found that responders compared to non-responders showed a relative increase in positive bias in the misclassification of facial expressions (however, there was no significant linear relationship between HAM-D Improvement and change in positive bias). This can be interpreted as an increased tendency to interpret ambiguous facial expressions as positive. Similar increases in positive bias have been reported in response to other antidepressant treatments and might contribute to symptom reduction by disrupting negative thought patterns [7, 20, 21, 23].

Using an fMRI task presenting happy versus fearful faces, we found that an early increase in positive bias in negative BOLD response in rACC, precuneus and angular gyrus was associated with improved mood outcomes. Pre-treatment activity in rACC [42], as well as increases in rACC volume [43], have been associated with response to TMS treatment. All three regions are part of the default mode network, which typically de-activates during the performance of simple cognitive tasks. Depression has been associated with reduced negative BOLD response of the DMN to external stimuli [34, 44], especially positive stimuli [45], which is hypothesised to drive rumination and reduced engagement with external stimuli. Our results suggest that TMS might normalise this deficit early on during treatment. The change in the precuneus was correlated to an increase in reaction time to happy faces in the gender discrimination task (i.e. attentional capture), showing that the neural changes translated into a concurrent increased positive bias in behaviour.

Response to TMS treatment was further related to positive bias in task-related functional connectivity (i.e. communication between different brain areas). HAM-D improvement was associated with increased positive bias in connectivity between the seed region in rACC and posterior parts of the DMN, sensorimotor and visual areas. In other words, patients who showed an early increase in task-related connectivity between rACC and several cortical areas during positive (compared to negative) faces showed better treatment outcomes. These connectivity changes correlated with concurrent increased positive bias in the gender discrimination task. Depression has been associated with reduced connectivity of rACC to posterior parts of the DMN [46], and sensorimotor regions [47]. Our data indicate that TMS treatment might normalise such impairments, potentially increasing processing of positive information and enhancing emotion regulation [46, 48].

The aim of our study was to investigate potential neurocognitive mechanisms of TMS treatment. By Week 2, HAM-D scores had already improved, so it is unclear whether the behavioural and neural changes in positive bias at Week 2 preceded that early clinical improvement. However, machine learning models indicated that the neural measures predicted clinical improvement at the end of treatment above and beyond the early clinical change. While this is not sufficient evidence to establish a causal relationship, this result indicates that the neural changes do not simply reflect early symptom reduction, and might represent a potential mechanism of TMS treatment. Future studies are needed to test if early changes in emotional processing are causally linked to clinical response.

Our results provide evidence suggesting that the neuropsychological theory of antidepressant action might generalise to TMS treatment. Antidepressant drugs have generally been reported to induce a positive bias in regions of the salience network such as dorsal ACC, insula and amygdala (see [49, 50] for review). Interestingly, we found no such effect in our study. While our study did not include direct comparison with a pharmacological intervention, this raises the possibility that the neural mechanisms may differ. While drugs might primarily affect the salience network, TMS might primarily affect the DMN, likely via negative functional coupling between DLPFC and subgenual ACC [13, 51–53].

Moreover, treatment response was not related to changes in the ventral striatum. Hypoactivity in response to rewards and positive stimuli has been linked to symptoms of depression, particularly to anhedonia [54–57]. Antidepressant drugs have been found to modulate activity in the ventral striatum [54, 58]. Regarding TMS treatment, pre-treatment connectivity of the striatum has been linked to clinical response [59–62]. Future studies could include reward learning tasks which might be more sensitive to striatal activity than emotional processing tasks.

This study has several limitations. First, the study did not include a control group (e.g. sham stimulation) which would allow for the quantification of placebo effects. Therefore, it cannot be ruled out that the observed changes might be related to placebo effects or differences in natural disease trajectory. Second, since symptoms had already improved substantially after 8 sessions, it is unclear whether changes in emotional processing preceded (and caused) clinical improvement. Third, many patients in our sample were taking a stable dose of psychoactive medication (which was controlled for in the analysis). Consequently, it cannot be ruled out that the observed effects might partially be related to interactions with medication. Fourth, the sample was heavily skewed towards women. Replication studies are necessary to assess the replicability and generalisability of our findings to more diverse populations (including men). The most important point to address in future research would be the addition of a control group (e.g. sham TMS). This would enable the contribution of placebo effects or natural recovery to be controlled for. Moreover, the inclusion of multiple earlier testing timepoints would help to establish when and how the change in positive bias unfolds over time. To investigate how the mechanisms of TMS treatment might differ from those of antidepressant drugs, additional treatment arms (drug/placebo) could be included to enable direct comparison.

## Supplementary information

Supplementary Material

## Data Availability

Analysis scripts reproducing all key results and figures are available at https://osf.io/fmhpt/. Group-level statistical maps from the fMRI analysis are available at https://identifiers.org/neurovault.collection:20528. Due to the clinically sensitive nature of our data, we are unable to share individual participant data.
